# Mortality of traumatic chest injury and its predictors across sub-saharan Africa: systematic review and meta-analysis, 2024

**DOI:** 10.1186/s12873-024-00951-w

**Published:** 2024-02-27

**Authors:** Ousman Adal, Abiyu Abadi Tareke, Eyob Ketema Bogale, Tadele Fentabil Anagaw, Misganaw Guadie Tiruneh, Eneyew Talie Fenta, Destaw Endeshaw, Amare Mebrat Delie

**Affiliations:** 1 Department of Emergency and Critical Care Nursing, College of Medicine and Health Sciences, Bahir Dar University, Bahir Dar, Ethiopia; 2 SLL project, COVID-19 vaccine/EPI Technical Assistant at West Gondar Zonal Health Department, Amref Health Africa in Ethiopia, Bole Sub City, Ethiopia; 3 Health Promotion and Behavioral Science Department, College of Medicine and Health Sciences, Bahir Dar University, Bahir Dar, Ethiopia; 4 Department of Health Systems and Policy, Institute of Public Health, College of Medicine and Health Sciences, University of Gondar, Gondar, Ethiopia; 5 Department of Public Health, College of Medicine and Health Science, Injibara University, Injibara, Ethiopia; 6 Department of Adult Health nursing, College of Medicine and Health Sciences, Bahir Dar University, Bahir Dar, Ethiopia

**Keywords:** Traumatic chest injury, Mortality, Sub-saharan Africa

## Abstract

**Introduction:**

Globally, chest trauma remain as a prominent contributor to both morbidity and mortality. Notably, patients experiencing blunt chest trauma exhibit a higher mortality rate (11.65%) compared to those with penetrating chest trauma (5.63%).

**Aim:**

This systematic review and meta-analysis aimed to assess the mortality rate and its determinants in cases of traumatic chest injuries.

**Methods:**

The Preferred Reporting Items for Systematic Reviews and Meta-Analyses (PRISMA) checklist guided the data synthesis process. Multiple advanced search methods, encompassing databases such as PubMed, Africa Index Medicus, Scopus, Embase, Science Direct, HINARI, and Google Scholar, were employed. The elimination of duplicate studies occurred using EndNote version X9. Quality assessment utilized the Newcastle-Ottawa Scale, and data extraction adhered to the Joanna Briggs Institute (JBI) format. Evaluation of publication bias was conducted via Egger’s regression test and funnel plot, with additional sensitivity analysis. All studies included in this meta-analysis were observational, ultimately addressing the query, what is the pooled mortality rate of traumatic chest injury and its predictors in sub-Saharan Africa?

**Results:**

Among the 845 identified original articles, 21 published original studies were included in the pooled mortality analysis for patients with chest trauma. The determined mortality rate was nine (95% CI: 6.35–11.65). Predictors contributing to mortality included age over 50 (AOR 3.5; 95% CI: 1.19–10.35), a time interval of 2–6 h between injury and admission (AOR 3.9; 95% CI: 2.04–7.51), injuries associated with the head and neck (AOR 6.28; 95% CI: 3.00–13.15), spinal injuries (AOR 7.86; 95% CI: 3.02–19.51), comorbidities (AOR 5.24; 95% CI: 2.93–9.40), any associated injuries (AOR 7.9; 95% CI: 3.12–18.45), cardiac injuries (AOR 5.02; 95% CI: 2.62–9.68), the need for ICU care (AOR 13.7; 95% CI: 9.59–19.66), and an Injury Severity Score (AOR 3.5; 95% CI: 10.6–11.60).

**Conclusion:**

The aggregated mortality rate for traumatic chest injuries tends to be higher in sub-Saharan Africa. Factors such as age over 50 years, delayed admission (2–6 h), injuries associated with the head, neck, or spine, comorbidities, associated injuries, cardiac injuries, ICU admission, and increased Injury Severity Score were identified as positive predictors. Targeted intervention areas encompass the health sector, infrastructure, municipality, transportation zones, and the broader community.

**Supplementary Information:**

The online version contains supplementary material available at 10.1186/s12873-024-00951-w.

## Introduction

Trauma remain as a persistent contributor to global morbidity and mortality [1]. It often manifests across multiple anatomical areas, prominently involving the chest [1]. Chest trauma, ranging from simple rib fractures to severe penetrating injuries affecting vital structures such as the heart or tracheobronchial system, is observed in nearly two-thirds of patients [2]. Globally, chest trauma ranks among the foremost causes of morbidity and mortality, particularly impacting the younger demographic. It holds the position as the third most prevalent injury worldwide, trailing only head and extremity injuries [3]. Thoracic injury-related mortality, the second highest following head injury, underscores the critical significance of initial management in these cases. Notably, 25% of all trauma-related deaths worldwide stem from chest trauma alone [4]. A retrospective cross-sectional study in Tanzania and Egypt revealed that chest (thoracic) trauma carries an overall mortality rate of 15–25%, surpassing that of patients with cardiac or tracheobronchial–esophageal injuries. This emphasizes the gravity of chest trauma within the spectrum of traumatic injuries [5].

According to a study conducted in Europe (Spain), various factors are associated with mortality due to chest trauma. These factors include age, severity of injury, associated brain injury, hemodynamic instability, the need for prehospital intubation (ICU), and injury and multi-organ failure [6]. Another study in Egypt showed that age, unconsciousness, shock, and the need for surgical intervention are predictors of chest trauma-related mortality [7]. The existence of concomitant injuries, such as haemothorax, pneumothorax, and hemopneumothorax, along with untreated vascular injuries, can contribute to the mortality of chest trauma if not timely managed [8]. According to a recent study in Ethiopia, the mortality rate of chest trauma was 27% in 2020 and 26% in 2023 [9]. A recent study conducted in Ethiopia revealed that late presentation beyond 6 h, patient age (> 50), penetrating injury, bilateral chest injury, associated extra-thoracic injury, and the need for ICU care were predictors of mortality following traumatic chest injuries [9, 10].

Most chest traumas are blunt. For instance, a study conducted in Pakistan revealed that 126 (63.3%) patients had blunt chest injuries, whereas 73 (36.6%) had penetrating chest injuries [11]. Previous studies in sub-Saharan Africa have indicated that road traffic accidents were the most common cause of blunt chest injuries, accounting for 83 (65.8%) patients. In contrast, gunshots were the leading cause of penetrating chest injuries, accounting for 41 (56.2%) cases [11]. Mortality was higher in blunt chest trauma (11.65%) than in penetrating chest trauma (5.63%) [12]. Similar to other sub-Saharan African countries, in Ethiopia, road traffic accidents (RTA) (44.5%) were the most common cause of chest trauma, followed by violence (34.9%). Although the authors sought studies on this subject, no study had been conducted on the pooled mortality of chest injuries in sub-Saharan Africa; rather, individual studies are available. Pooling data from multiple studies through systematic reviews and meta-analyses can provide a more comprehensive and reliable understanding of overall trends and outcomes. Therefore, this systematic review and meta-analysis examine the pooled mortality of traumatic chest injuries and their determinants throughout sub-Saharan Africa.

### Research questions

What is the pooled mortality rate of traumatic chest injury in sub-Saharan Africa?

What are the pooled factors contributing to the mortality rate of chest injury across sub-Saharan Africa?

## Methods

### Protocol and registration

The findings presented in this review adhere to the guidelines defined in the Preferred Reporting Items for Systematic Review and Meta-Analysis (PRISMA) [13]. The protocol for this review has been prospectively registered with the International Prospective Register of Systematic Reviews (PROSPERO) under the registration number CRD42023485003.

### Inclusion and exclusion criteria

All types of studies (both published and unpublished) reporting the mortality of chest trauma and published in English across sub-Saharan Africa were included. The findings were not restricted to a specific study period. All age groups in all health facilities pre hospital (on scene death, health post, clinic, and health center), all types of hospitals (primary, general and comprehensive specialized) and all department (emergency, ward, and intensive care unit, pediatric and neonatal unit) were included. Citations without abstracts and/or full-text and anonymous reports, editorials, and qualitative studies were excluded from the analysis. Studies that solely focused on specific intrathoracic injuries, such as cardiac injury, were also excluded.

### Search strategy and selection criteria

The authors employed various advanced searching techniques between November 21−30/2023 to conduct this review across relevant databases, including PubMed, Africa Index Medicus, Science Direct, Scopus, Embase, HINARI, and Google Scholar. In addition, the authors accessed the online library repositories of Addis Ababa University and Bahir Dar University. Moreover, the authors searched for references in each article within the reviewed studies that were relevant to the objective of this review and meta-analysis. The main search terms in PubMed comprised ‘Mortality’ OR ‘Death’ OR ‘Outcome’ AND ‘Epidemiologic Factors’ OR ‘Predictor’ OR ‘Factor’ OR ‘Associated Factor’ AND ‘Thoracic Injuries’ OR ‘Thoracic Trauma’ OR ‘Chest Trauma’ OR ‘Chest Injury’ AND ‘Africa South of the Sahara.

### Quality assessment and data abstraction procedures

All identified studies from the database were imported into the citation manager, EndNote version X9, to eliminate duplicate studies and process further. Five authors (OA, DE, EK, TF, and MG) independently reviewed and screened the titles and abstracts of the identified studies. Any disagreements that arose were resolved through discussion with the fourth author (OA) on the basis of pre-established article selection criteria. To assess the quality of each study, the authors used the Newcastle– Ottawa Scale [14], which was adapted for the systematic review to evaluate the quality of studies [15]. The assessment considered three key aspects: (1) Selection (with a maximum of 4 stars), (2) Comparability (with a maximum of 3 stars), and (3) Outcome (with a maximum of 2 stars). Each original article was appraised by each author individually. In the case of discrepancies between the authors, an agreement was reached by averaging the scores provided by the five authors. The score of each study was calculated on a scale from 0 to 10 for cross-sectional studies and zero to nine for cohort and case-control studies. A score > 6 was considered ‘good’ and included in the study [15]. Additionally, publication bias was evaluated using Egger’s regression test, funnel plot, and sensitivity analysis. Noteworthy is the fact that the reliability of these instruments across the diverse range of studies under consideration demonstrated a commendable consistency, as reflected by Cronbach alphas spanning from 7.5 to 8.9. Furthermore, the rigorous validation process undertaken by the three experts encompassed a comprehensive assessment of each study within the review.

### Outcome measurement

The main outcome is mortality from thoracic (chest) trauma, defined as the proportion of all traumatic chest injury patients who died among the studies included in this review. This proportion was calculated by dividing the total number of patients who died from traumatic chest injuries by the total number of traumatic chest injury patients included in this review study, multiplied by 100. The authors used the adjusted odds ratio as an outcome measure to identify predictors of mortality among patients with traumatic chest injuries.

### Data extraction and analysis

**Fig. 1:**
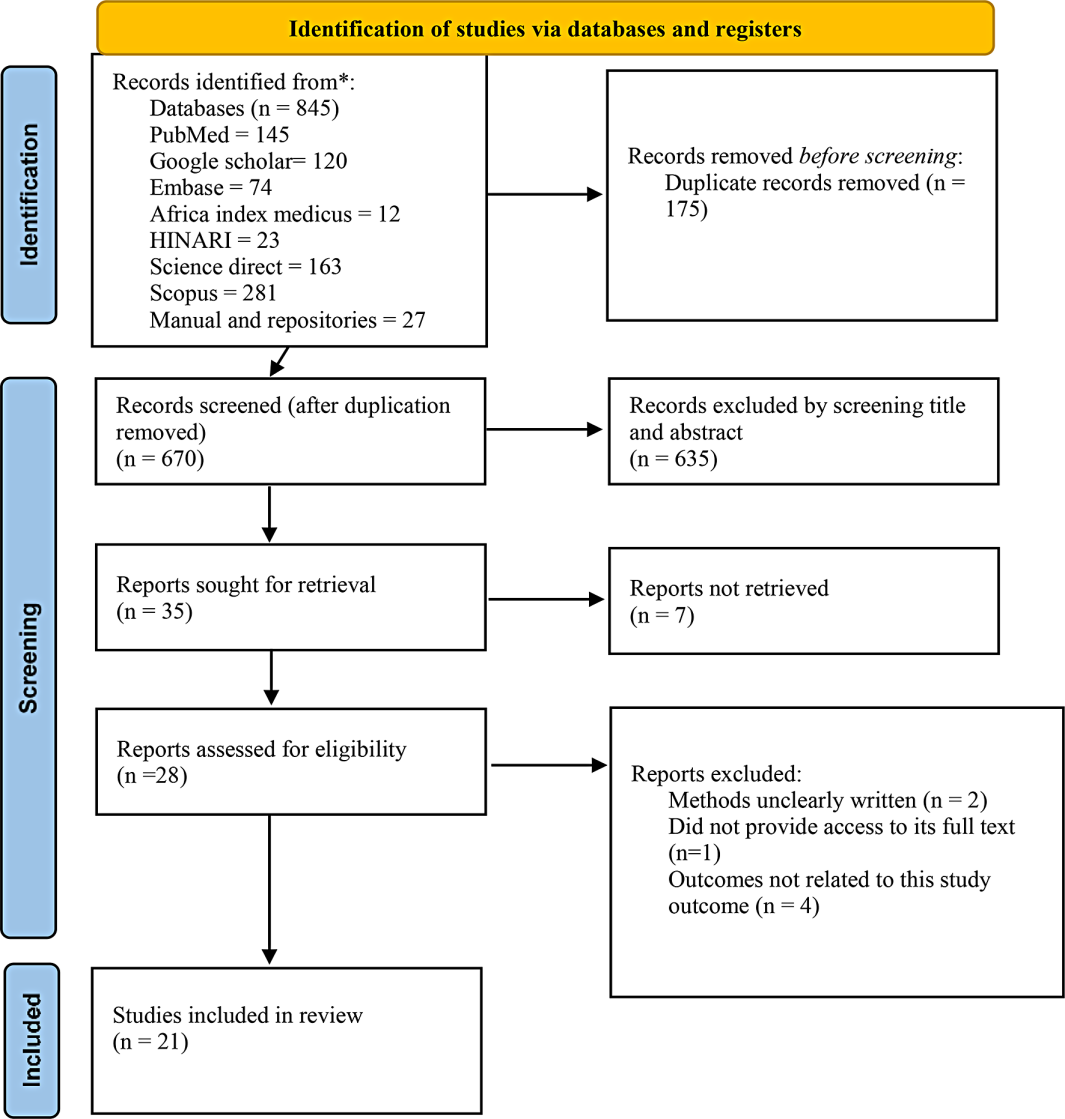
A flowchart showing the sequence of study selection using PRISMA

The data were extracted using the standard format adapted from the Joanna Briggs Institute (JBI) data extraction format [16]. The four authors independently extracted relevant data using this format. In situations where disagreements arose between authors during the data extraction procedure, they were resolved through discussion and consensus. The data extraction format included the primary author’s name, publication year, country, study design, sample size, sampling technique, and mortality with a 95% confidence interval (CI), the logarithm of proportion, and associated factors of mortality with a 95% CI. The statistical software STATA version 17 was used for the meta-analysis. Pooled analysis was conducted using the random-effects Dersimonian– Laird model [17]. The level of heterogeneity between the studies was measured using the I-squared statistic. Trim-and-fill analyses were also performed to assess publication bias and heterogeneity. Moreover, sensitivity analysis was conducted. The Preferred Reporting Items for Systematic Reviews and Meta-Analyses (PRISMA) checklist was used for data presentation [13] (Fig. 1).

## Results

### Search results and characteristic of the reviewed studies

The authors initially identified 845 original articles from various databases, including PubMed, Africa Index Medicus, Scopus, Embase, Science Direct, and HINARI, along with manual searches. After eliminating 175 duplicate articles, 670 remained. Following the screening of titles and abstracts, 635 studies were excluded due to their irrelevance to the current study. The remaining 35 studies underwent further evaluation, and only 28 met the inclusion criteria. Seven articles were subsequently excluded for various reasons: one lacked full-text access [18], two had unclearly written methods [19, 20], and the remaining four had outcomes unrelated to this study [21–24]. Finally, 21 studies were included in this systematic review and meta-analysis [4, 25–44] (Fig. 1).

Twenty-one eligible studies included in the meta-analysis were published between 1981 and 2023. Of these 21 published original studies that reported the proportion of mortality, four were conducted in Ethiopia [26, 29, 39, 40], and the majority of studies (nine) were conducted in Nigeria [4, 25, 27, 28, 33–37]. The other studies were conducted in Tanzania (three) [30, 31, 43], South Africa (two) [41, 42], Cameroon (one) [32], Senegal (one) [44], and Sudan (one) [38]. Most studies were conducted with a cross-sectional design (thirteen) [4, 25–27, 29, 31, 32, 36, 39–42, 44], and the fewest were conducted via prospective cohort studies (eight) [28, 30, 33–35, 37, 38, 43]. The sample size of each study ranged between 40 and 442. In this review study, 3939 patients (cumulative sample size) were included.

### Causes, mechanisms, and associated extra thoracic injuries for each study among traumatic chest injury patients across sub-Saharan Africa

Most injuries resulted from road traffic accidents (RTA), with blunt thoracic injuries being the dominant mechanism. Most patients with chest trauma had associated head and neck injuries, followed by injuries to the extremities. The highest mortality was reported in Ethiopia (27.6%) [26]. whereas the lowest mortality was reported in Nigeria (1.1%) [27] (Table 1).

**Table 1:** Causes, mechanisms, and associated extra thoracic injuries among patients with traumatic chest injury across sub-Saharan Africa

Country with publication year	RTA %	firearm injury %	Stab%	fall down%	Mechanism		Associated extra thoracic Injuries							Reference
					Blunt %	Penetrating %	Hand and neck%	Abdomen%	Spinal%	Limb%	Sample size%	Study design	Mortality%	
Ethiopia 2020	44.5	34.9	2.2	18.4	64.1	35.9	27.6	34.9	9.9	48.4	192	Cross-sectional	27.6	[26]
Nigeria2004	38.46	45.76	10.34	5.44	38.46	61.53	2.6	12.8	1.6	20.5	78	Cross-sectional	2.56	[25]
Nigeria 1981	73.1	8	4	14.9	82	18	1.5	15	1.3	17	145	Cross-sectional	9.7	[4]
Senegal 1995							2	33			179	Cross-sectional		[44]
south Africa2006	16	1.9	71.1	10.8	17.9	82.1	4.5	60	0.2		117	Cross-sectional	15.6	[41]
Nigeria2014	0.3	12.6	21.2		65.1	34.9	13.2	15	1.2	5.8	149	Cohort	5.3	[28]
Nigeria2018	0.55	15.8	2.9	2.7	69.7	30.3	2.8	3.4	0.9	3.4	442	Cross-sectional	1.1	[27]
Ethiopia2023	33.3	6	42.7	18	53.4	46.6	20.4	38.8	3	3	103	Cross-sectional	3.1	[29]
south Africa2022											40	Cross-sectional	3	[42]
Tanzania2011	50.7	7	14	16	72.7	27.3	33.3	5.3		26.7	150	Cohort	3.3	[30]
Tanzania2010	72.3	9	17	5	75.6	23.4	17	13	4	20	119	Cohort	24.2	[43]
Tanzania2023	65.7	5	4	20.3	95.58	4.42	60.5	8.8	21.15	48.2	114	Cross-sectional	21	[31]
Cameroon 2010	63.8				65.3	34.7					354	Cross-sectional	7.6	[32]
Nigeria2007	70.7	7.8	14	6.9	72.2	27.8	28.1	21.1	1	33.3	198	Cohort	4.5	[33]
Ethiopia2021	52.5		28.6	19.9	55.8	44.2	21.7	18.1	6.7	22.2	419	Cross-sectional	26	[40]
Ethiopia 2022	34.5	25	20.75	19.75	57.25	42.75	17	30	1	13.75	422	Cross-sectional	7.2	[39]
Nigeria2012	56.9	8	33.3	1.8	86.8	13.6	29.6	9.9	1.1	35.8	114	Cohort	11	[34]
Nigeria2015	10	3	4	13	77.4	22.6	23.2	10.4	1.2		256	Cohort	3.1	[35]
Nigeria2021	53.7	21	18.2	4.9	66.7	33.3	24	11.2	0.4		162	Cohort	5.9	[37]
sudan2015	73.3	0.7	8	4	73.3	26.7	13.3	19.3	0.7	37.3	150	Cohort	2.1	[38]
Nigeria 2012	52	25	22.6	1.4	57	43	12.9	15.2	0.2	11.2	73	Cross-sectional	2.7	[36]

## Meta-analysis results

### The pooled mortality of traumatic chest injury

**Fig. 2:**
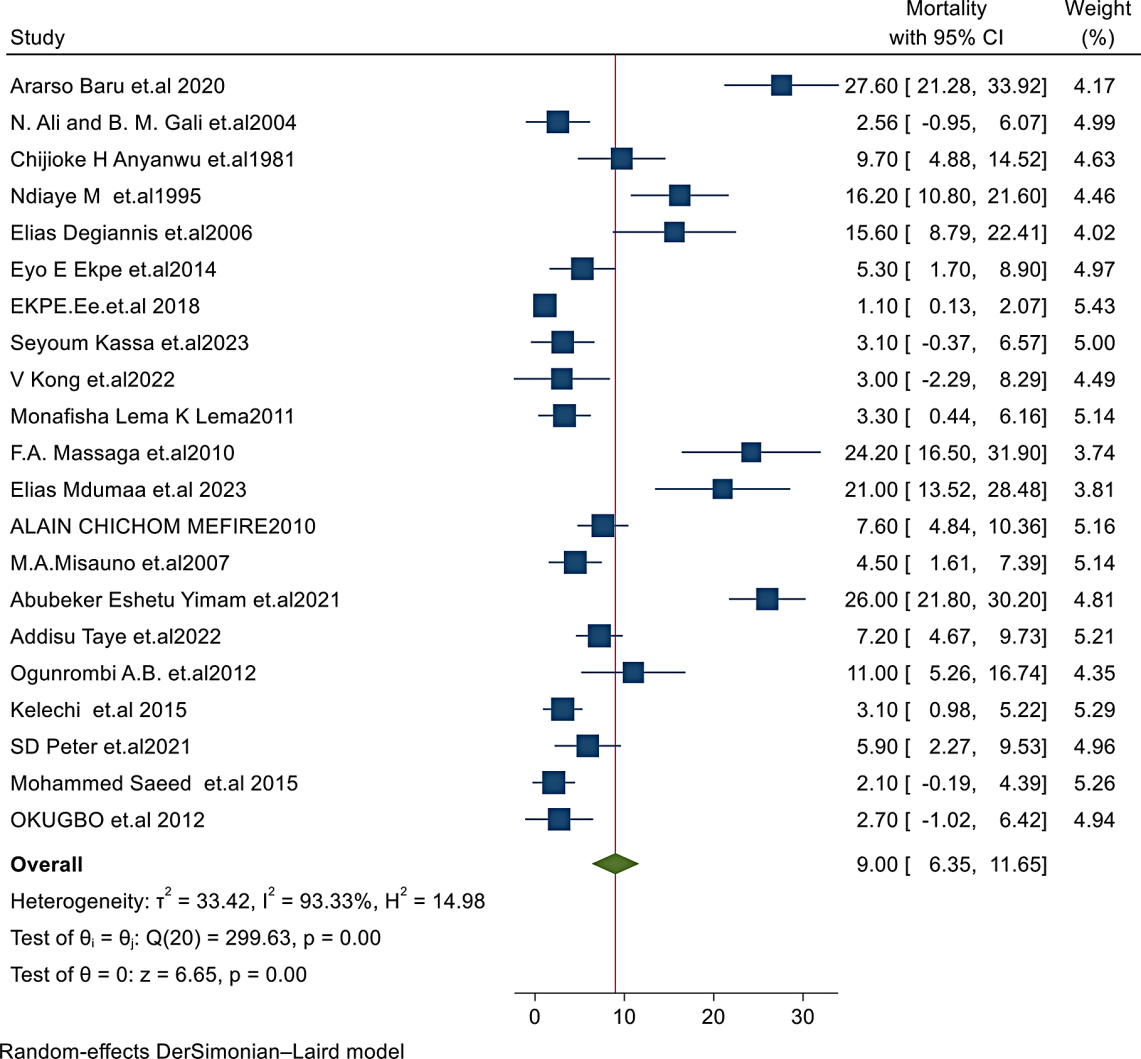
A forest plot for the pooled mortality rate of patients with traumatic chest injuries across sub-Saharan Africa using the random-effects dersimonian-laird model

In this reviewed study, the pooled mortality rate of patients who suffered from chest trauma, using the random-effects Dersimonian-Laird model, was found to be approximately 9% (95% CI: 6.35, 11.65) (Fig. 2).

### Heterogeneity results

The heterogeneity between studies in this review was high (I^2^ = 93.33%, t^2^ = 33.42%, t = 6.65) (Fig. 2), and it was significant (*P* < 0.001) with a 95% CI. The source of the high I^2^ is not identified. However, an increase in heterogeneity is expected in the meta-analysis of the proportion of mortality in different countries and study designs, and the results could be interpreted sequentially and with caution. Moreover, the study tested a wide prediction interval, which was a direct and easily interpretable indicator compared to the CI, suggesting high heterogeneity.

### Publication bias and sensitivity test results

**Fig. 3:**
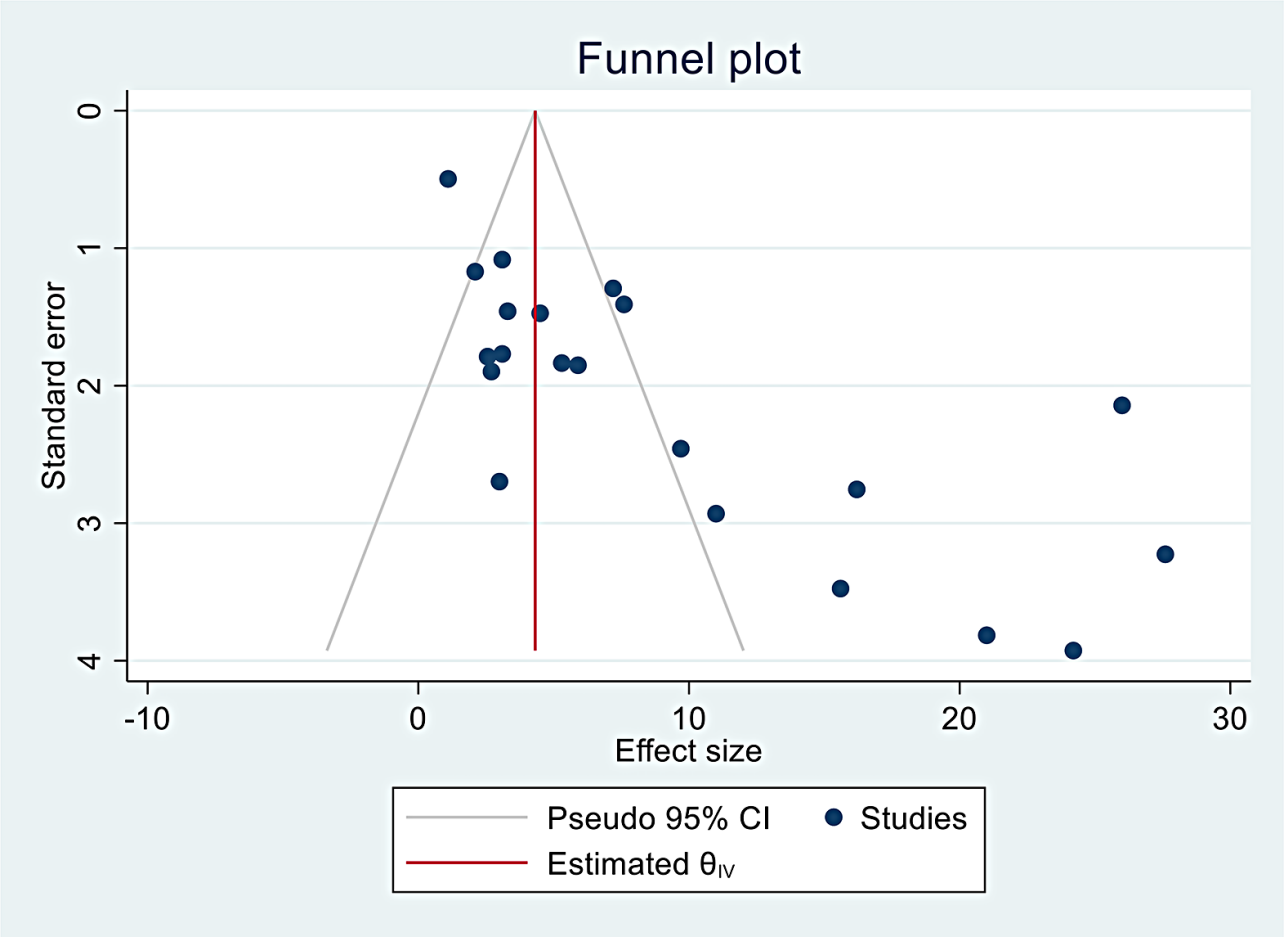
Publication bias with funnel plot of pseudo 95% CI on pooled mortality on traumatic chest injury across sub-Saharan Africa

**Fig. 4:**
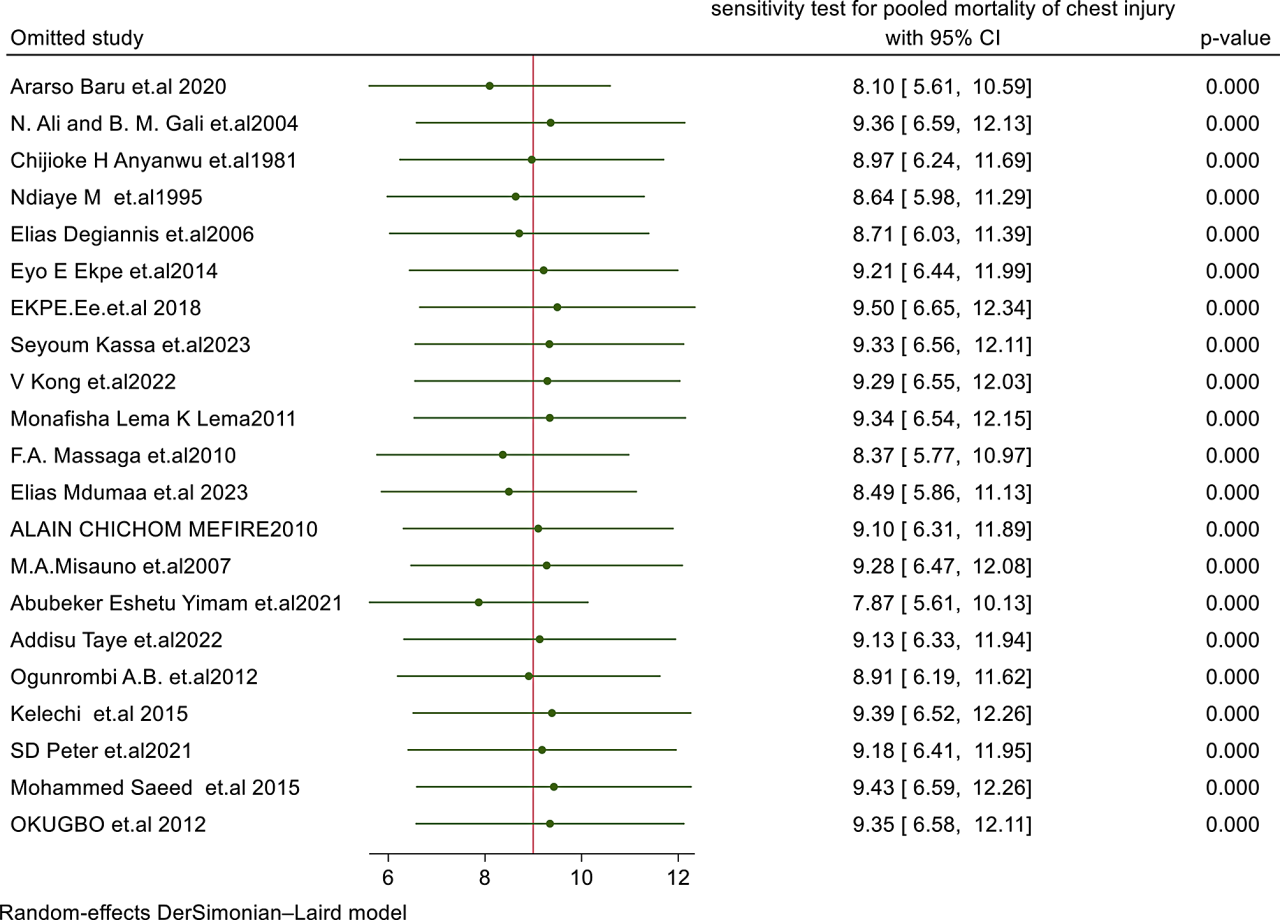
Sensitivity analysis for pooled mortality of traumatic chest injury

Publication bias was assessed using Begg’s test (*p* = 0.005) and Egger’s regression test (*p* < 0.001), both of which showed significant publication bias. The study also observed asymmetry in the funnel plot (Fig. 3). Trim and fill analyses were also performed to address publication bias and heterogeneity. Moreover, sensitivity analysis was conducted, and all estimates were within the confidence interval limits (Fig. 4), showing that no individual study contributed to publication bias. As a result, none of the studies were excluded from the final meta-analysis.

### Subgroup analysis for mortality

Subgroup analysis by country showed the following percentages: Ethiopia 15.76% (95% CI: 4.62, 26.90), Nigeria 4.47% (95% CI: 2.52, 6.41), Tanzania 15.84% (95% CI: 0.827, 30.86), South Africa 9.11% (95% CI: 3.233, 21.45), Sudan 2.1%, Senegal 16.2%, and Cameroon 7.6%. In this reviewed study, the highest pooled mortality rates were reported in Tanzania (15.84%) and Ethiopia (15.76%). Subgroup analysis by study design showed cross-sectional studies at 10.65% (95% CI: 6.38, 14.92) and cohort studies at 6.04% (95% CI: 3.36, 8.71). Subgroup analysis by publication year revealed rates of 7.27% (95% CI: 4.81, 9.73) for 1981−2015 and 11.50% (95% CI: 5.28, 17.72) for 2016−2023 (Table 2).

**Table 2:** Subgroup analysis of patients with traumatic chest injuries based on country, study design, and publication year across sub-Saharan Africa

	Variables	Response	number of studies	Pooled mortality with 95% CI	I2 (p-value)
1	Country	Ethiopia	4	15.76% (95% CI: 4.62, 26.90)	97.12% (< 0.001)
		Nigeria	9	4.46% (95% CI: 2.52, 6.41)	75.62% (< 0.001)
		Tanzania	3	15.84% (95% CI: 0.83, 30.86)	94.86% (< 0.001)
		South Africa	2	9.11% (95% CI: 3.23, 21.45)	87.81% (< 0.001)
		Cameroon	1	7.6% (4.84, 10.36)	-
		Senegal	1	16.20% (95% CI: 10.80, 21.59)	-
		Sudan	1	2.10% (95% CI: 0.19, 4.39)	-
2	Study design	Cross-sectional	13	10.65% (95% CI: 6.38, 14.92)	95.43% (< 0.001)
		cohort	8	6.04% (95% CI: 3.36, 8.71)	81.18% (< 0.001)
3	Publication year	1981–2015	13	7.27% (95% CI: 4.81, 9.73)	84.01% (< 0.001)
		2016 − 223	8	11.50% (95% CI: 5.28, 17.72)	96.82% (< 0.001)

Note: CI, confident interval

### Factors associated with the mortality among patients with traumatic chest injury across sub-saharan Africa

Out of the total studies retrieved, nine factors were found to positively affect the mortality of traumatic chest injuries. The pooled effect of four studies [26, 34, 39, 40] revealed that individuals aged over 50 had 3.5 times higher odds (AOR 3.5; 95% CI: 1.19, 10.35) of mortality compared to those under 20. Similarly, the combined findings of three studies [26, 39, 40] indicated that a time interval between injury and admission of 2–6 h was associated with 3.9 times higher odds of mortality (AOR 3.9; 95% CI: 2.04, 7.51) compared to admission within less than 2 h. Additionally, the amalgamated results of three studies [26, 31, 40] demonstrated that associated injuries with the head and neck carried six times higher odds of mortality (AOR 6.28; 95% CI: 3.00, 13.15) than their counterparts. Furthermore, the combined effect of two studies [26, 40] showed that associated spinal injuries were associated with 7.8 times higher odds of mortality (AOR 7.86; 95% CI 3.02, 19.51) compared to cases without spinal injuries. The pooled effect of two studies [29, 30] indicated that the presence of comorbidities had 5.2 times higher odds of mortality (AOR 5.24, 95% CI 2.93, 9.40) compared to cases without comorbidities. The pooled effect of three studies [30, 39, 40] showed that any associated injuries had 7.6 times higher odds of mortality (AOR 7.59; 95% CI: 3.12, 18.45) compared to patients without any associated injury. The pooled effect of two studies [26, 44] showed that associated cardiac injuries were associated with five times higher odds (AOR 5.02; 95% CI: 2.62, 9.68) as predictors of mortality compared to cases without cardiac injury. The combined results of two studies [30, 40] showed that the need for ICU care had 13.7 times higher odds (AOR 13.7; 95% CI 9.59, 19.66) as predictors of mortality compared to patients who did not require ICU care. Furthermore, the combined effect of three studies [30, 34, 40] demonstrated that the Injury Severity Score (ISS) was associated with 3.5 times higher odds (AOR 3.5; 95% CI: 10.6, 11.60) as predictors of mortality among patients with traumatic chest injuries across sub-Saharan Africa (Table 3).

**Table 3:** Factors associated with the mortality among patients with traumatic chest injury across sub-Saharan Africa, 2023

	Factor	No of included studies	Pooled AOR (95% CI)	I^2^ (p-value)	Reference category
1	Age > 50	4	3.51 ( 1.19, 10.35)	84.25% (0.02)	Age < 20
2	Time between injury and admission 2–6 h	3	3.91 (2.04, 7.51)	0.00% (< 0.001)	< 2 hors
3	Associated injury head and neck	3	6.28 (3.00, 13.15)	25.88% (< 0.001)	Extremities
4	Spinal injury (yes, No)	2	7.86 (3.02, 19.51)	0.00% (< 0.001)	No
5	Comorbidity	2	5.24 (2.93, 9.40)	0.00% (< 0.001)	No
6	Associated injury (yes, No	3	7.59 (3.12, 18.45)	66.23% (< 0.001)	No
7	Cardiac injury	2	5.02 (2.62, 9.68)	0.00% (< 0.001)	No
8	ICU needed	2	13.73 (9.59, 19.66)	0.00%(< 0.001)	No
9	Injury severity score (increased)	3	3.5 (10.6, 11.60)	88.21%(0.04)	Decreased

Note: AOR, adjusted odds ratio, CI, confident interval

## Discussion

This systematic review and meta-analysis examine the pooled mortality of traumatic chest injuries and their determinants throughout sub-Saharan Africa. In this meta-analysis, the collective mortality rate among patients who underwent sustained chest trauma was precisely determined using the random-effects Dersimonian-Laird model. The identified mortality rate stands at 9%, with a 95% confidence interval ranging from 6.35 to 11.65%. To put it into perspective, it suggests that, on average, out of one hundred of patients with traumatic chest injuries, approximately nine individuals succumbed to the condition. Furthermore, this constitutes a significant concern, as it represents a prevalent cause of death in sub-Saharan Africa.

This prevalence is notably higher than the 0.16% reported in the reviewed study conducted in the United States [45]. The disparity in the standard of medical care and the overall level of socioeconomic development in high income countries may serve as a contributing factor to this observed difference [46]. This study, however, is consistent with the previous Iranian study conducted in 2018, which found that 10.07% of patients with chest injuries died [47].

The pooled mortality rate of this study is also higher than the systematic review and meta-analysis conducted in the United Kingdom in 2012 and 2015, which showed mortality rates of 5.3% and 6%, respectively [48, 49].The difference might be attributed to the low level of security in sub-Saharan African countries, as well as the prevalence of trauma and warfare [50, 51]. The significant struggles in sub-Saharan Africa have all contributed to a higher mortality rate in traumatic chest injuries [18, 29]. This implies that the mortality of patients following chest injury in sub-Saharan Africa is considerably greater than in high income countries, with no change in trends from 2012 to 2015 [51, 52]. This underscores the need for action-based interventions focused on health system improvement and a trauma reduction plan.

In addition, the prevalence of road traffic accidents (RTA) in sub-Saharan Africa (SSA) is a major challenge, contributing significantly to chest trauma [52]. For example, recent studies in Ethiopia and Tanzania revealed that RTAs accounted for 44.5% and 50.7%, respectively, with mortality following chest trauma reaching 27.6% [50]. Moreover, in many SSA countries, a delay of 2–6 h in seeking medical care is common due to a lack of transportation (ambulances) and poor road infrastructure. This is often linked to a scarcity of emergency care services and adequately qualified/trained trauma care professionals [50, 52]. Many SSA countries face challenges such as inadequate emergency teams (paramedics, surgeons, and nurses), insufficient equipment (first aid kits), limited prehospital care, and a lack of equipped trauma centers [53]. For instance, Ethiopia lacks paramedics nationwide, has no trauma center at the regional level, and has a shortage of chest surgeons [50]. Additional issues include the absence of trauma courses in some major universities and difficulties in accessing remote areas, making it challenging for emergency medical services (EMS) to provide timely assistance. Disparities in services are also evident regionally, with similar challenges observed across many SSA countries [52, 53].

Concerning the predictors of mortality in individuals with traumatic chest injuries, the cumulative impact of factors in this examined study indicates an increased mortality rate associated with the following listed factors. These included patients aged over 50, those facing a 2–6 h admission delay, individuals with associated head and neck injuries, patients with spinal injuries, those with comorbidities, individuals with additional injuries, those necessitating ICU care, and an escalation in the injury severity score. All of these factors were found to be associated with an increased likelihood of mortality in patients with traumatic chest injuries. Advancing age, coupled with concurrent injuries like spinal, head, and neck trauma, as well as the presence of comorbidities, often heightens the susceptibility of patients to complications such as shock and multiple organ failure. Consequently, this elevated risk significantly amplifies the likelihood of mortality following traumatic chest injury [5, 48, 49]. By directing our attention towards these specific factors or predictors, we can elevate our overall state of preparedness and improve response mechanisms. This approach ensures prompt and effective assistance for individuals confronted with life-threatening conditions. Through a comprehensive understanding and consideration of these elements, we strengthen our ability to provide timely and efficient support in critical situations.

### Limitation of the study

In this study, the authors utilized PubMed with the query “(Mortality/death/outcome) AND (epidemiological factors/predictor/factor/associated factor) AND (thoracic injuries/thoracic trauma/chest trauma/chest injury) AND (Africa South of Sahara).” Despite the first question not requiring the third string, its inclusion, indicated by “AND,” excluded studies lacking these terms but containing “mortality.” To address this limitation, the authors expanded their searches to include Africa Index Medicus, HINARI, Science Direct, Scopus, Embase, and manual repositories for comprehensive coverage.

## Conclusion

The pooled mortality of traumatic chest injury tends to be higher in sub-Saharan Africa. Patients aged over 50, those with delayed admission of 2–6 h, patients with associated head and neck injuries, patients with spinal injuries, the presence of comorbidities, patients with any associated injuries, patients requiring ICU admission, and increments in the injury severity score had positive predictors of mortality related to traumatic chest injury.

### Implication of the study

This finding highlights a higher mortality rate associated with traumatic chest injuries in sub-Saharan Africa. The esteemed authorities are urged to take proactive measures in critical sectors, specifically in health facilities, municipal infrastructure, and community engagement. This intervention is crucial to mitigate the mortality rates linked to traumatic chest injuries. The targeted intervention areas include the health sector, infrastructure, municipality, transportation zones, and the community at large.

### Electronic supplementary material

Below is the link to the electronic supplementary material.

Supplementary Material 1: Newcastle-Ottawa Scale adapted for cross-sectional studies for mortality of traumatic chest injurt

Supplementary Material 2: PRISMA 2020 flow diagram for new systematic reviews which included searches of databases and registers only

## Data Availability

All data generated or analyzed during this study are included in the manuscript or supplementary information.

## Abbreviations

ICU: Intensive care Unit
ISS: Injury severity score
JBI: Joanna Briggs Institute
PRISMA: Preferred Reporting Items for Systematic Review and Meta-Analysis
RTA: Road traffic accident
SSA: Sub-Sahara Africa

## References

[1] Baker E, Xyrichis A, Norton C, Hopkins P, Lee G (2018). The long-term outcomes and health-related quality of life of patients following blunt thoracic injury: a narrative literature review. Scand J Trauma Resusc Emerg Med.

[2] Ludwig C, Koryllos A (2017). Management of chest trauma. J Thorac Disease.

[3] Elbaih AH (2017). Patterns and management of chest injuries patients and its outcome in Emergency Department in Suez Canal University Hospital, Egypt. Med Sci.

[4] Anyanwu CH, Swarup AS (1981). Chest trauma in a developing country. Ann R Coll Surg Engl.

[5] Azarhomayoun A, Aghasi M, Mousavi N, Shokraneh F, Vaccaro AR, Mirzaian AH (2018). Mortality rate and predicting factors of traumatic thoracolumbar spinal cord injury; a systematic review and meta-analysis. Bull Emerg Trauma.

[6] Barea-Mendoza JA, Chico-Fernández M, Quintana-Díaz M, Pérez-Bárcena J, Serviá-Goixart L, Molina-Díaz I (2022). Risk factors associated with mortality in severe chest trauma patients admitted to the ICU. J Clin Med.

[7] Elkhonezy BA, Abdelmoaty HM, Gamil IK (2021). Factors improve outcome of penetrating chest trauma. Egypt J Hosp Med.

[8] Adem A, Ilagoa R, Mekonen E. Chest injuries in Tikur Anbessa hospital, Addis Ababa: a three year experience. East Cent Afr J Surg. 2001;6(1).

[9] Yimam AE, Mustofa SY, Aytolign HA (2021). Mortality rate and factors associated with death in traumatic chest injury patients: a retrospective study. Int J Surg Open.

[10] Baru A, Weldegiorgis E, Zewdu T, Hussien H (2020). Characteristics and outcome of traumatic chest injury patients visited a specialized hospital in Addis Ababa, Ethiopia: a one-year retrospective study. Chin J Traumatol.

[11] Mazcuri M, Ahmad T, Abid A, Thapaliya P, Ali M, Ali N. Pattern and outcome of thoracic injuries in a busy tertiary care unit. Cureus. 2020;12(10). 10.7759/cureus.11181 PMC7593122 33133801

[12] Narayanan R, Kumar S, Gupta A, Bansal VK, Sagar S, Singhal M (2018). An analysis of presentation, pattern and outcome of chest trauma patients at an urban level 1 trauma center. Indian J Surg.

[13] Liberati A, Altman D, Tetzlaff J, Mulrow C, Gøtzsche P, Ioannidis J et al. The PRISMA statement for reporting systematic reviews and meta-analyses of studies that evaluate healthcare interventions. BMJ. 2009;339. 10.1136/bmj.b2700 PMC2714672 19622552

[14] Luchini C, Stubbs B, Solmi M, Veronese N (2017). Assessing the quality of studies in meta-analyses: advantages and limitations of the Newcastle Ottawa Scale. World J Meta-Analysis.

[15] Adegboye VO, Ladipo JK, Brimmo IA, Adebo AO (2002). Blunt chest trauma. Afr J Med Med Sci.

[16] Zeng X, Zhang Y, Kwong JS, Zhang C, Li S, Sun F (2015). The methodological quality assessment tools for preclinical and clinical studies, systematic review and meta-analysis, and clinical practice guideline: a systematic review. J evidence-based Med.

[17] IntHout J, Ioannidis JP, Borm GF (2014). The Hartung-Knapp-Sidik-Jonkman method for random effects meta-analysis is straightforward and considerably outperforms the standard DerSimonian-Laird method. BMC Med Res Methodol.

[18] Asfaw M, Aberra M (2005). A prospective analysis of thoracic injuries in Harar, Hiwot Fana hospital. Ethiop Med J.

[19] Gueye SN, Conty CR (1969). [Death due to chest injuries in traffic accidents in Dakar (results of 35 personally performed autopsies)]. Bull Soc Med Afr Noire Lang Fr.

[20] Kithuka CM, Ntola VC, Sibanda W, Afr (2023). J Surg.

[21] Adegboye VO, Ladipo JK, Adebo OA, Brimmo AI (2002). Diaphragmatic injuries. Afr J Med Med Sci.

[22] Adegboye VO, Osinowo O, Adebo OA (2003). Bronchiectasis consequent upon prolonged foreign body retention. Cent Afr J Med.

[23] Adenipekun A, Campbell OB, Oyesegun AR, Elumelu TN (2002). Radiotherapy in the management of early breast cancer in Ibadan: outcome of chest wall irradiation alone in clinically nodes free axilla. Afr J Med Med Sci.

[24] Adeoye PO, Salami MA, Oyemolade TA, Adegboye VO, CIVILIAN VASCULAR INJURIES IN AN URBAN AFRICAN REFERRAL INSTITUTION (2013). East Afr Med J.

[25] Ali N, Gali B (2004). Pattern and management of chest injuries in Maiduguri, Nigeria. Ann Afr Med.

[26] Baru A, Weldegiorgis E, Zewdu T, Hussien H (2020). Characteristics and outcome of traumatic chest injury patients visited a specialized hospital in Addis Ababa, Ethiopia: a one-year retrospective study. Chin J Traumatol.

[27] Ekpe EE, Etta O, Akpan AF (2018). Pattern of chest injuries and treatment outcome in a Nigerian teaching hospital. World J Biomed Res (Online).

[28] Ekpe EE, Eyo C (2014). Determinants of mortality in chest trauma patients. Nigerian J Surg.

[29] Kassa S, Aregawi Y, Genetu A, Gullilat D (2023). Exploring chest trauma: a Comprehensive Analysis of Injury characteristics, clinical outcomes, and management strategies in a Tertiary Care setting. Archives Infect Dis Therapy.

[30] Lema MK, Chalya PL, Mabula JB, Mahalu W (2011). Pattern and outcome of chest injuries at Bugando Medical Centre in Northwestern Tanzania. J Cardiothorac Surg.

[31] Mduma E, Chugulu S, Msuya D, Sakita F (2023). Pattern, management, and outcomes of chest Injury at Kilimanjaro Christian Medical Centre. East Afr Health Res J.

[32] Mefire AC, Pagbe JJ, Fokou M, Nguimbous JF, Guifo ML, Bahebeck J (2010). Analysis of epidemiology, lesions, treatment and outcome of 354 consecutive cases of blunt and penetrating trauma to the chest in an African setting. S Afr J Surg.

[33] Misauno M, Sule A, Nwadiaro H, Ozoilo K, Akwaras A, Ugwu B (2007). Severe chest trauma in Jos.

[34] Ogunrombi A, Onakpoya U, Ekrikpo U, Adesunkanmi A, Adejare I. The pattern and outcome of chest injuries in South West Nigeria. Annals Afr Surg. 2012;9(2).

[35] Okonta KE (2015). Traumatic chest injury in children: a single thoracic surgeon’s experience in two Nigerian tertiary hospitals. Afr J Paediatr Surg.

[36] Okugbo S, Okoro E, Irhibogbe P (2012). Chest trauma in a regional trauma centre. J West Afr Coll Surg.

[37] Peter S, Ozoilo K, Isichei M, Ale F, Njem J, Ojo E (2021). Severe chest injury revisited-an analysis of the Jos University Teaching Hospital Trauma Registry. Niger J Clin Pract.

[38] Saeed AY, Hamza AA, Ismail OM (2015). Pattern and management outcome of chest injuries in Omdurman Teaching Hospital Sudan. Global J Med Res.

[39] Taye A, Mersha L, Kindie W. Magnitude of chest trauma mortality and associated factors among adult patients admitted at University of Gondar Comprehensive Specialized Hospital, North West Ethiopia, 2019. 2022.

[40] Yimam AE, Mustofa SY, Gebregzi AHk, Aytolign HA (2021). Mortality rate and factors associated with death in traumatic chest injury patients: a retrospective study. Int J Surg Open.

[41] Degiannis E, Loogna P, Doll D, Bonanno F, Bowley DM, Smith MD (2006). Penetrating cardiac injuries: recent experience in South Africa. World J Surg.

[42] Kong V, Cheung C, Buitendag J, Rajaretnam N, Varghese C, Elsabagh A (2022). Management of penetrating thoracic trauma with retained knife blade: 15-year experience from a major trauma centre in South Africa. Ann R Coll Surg Engl.

[43] Massaga FA, McHembe M (2010). The pattern and management of Chest Trauma at Muhimbili National Hospital; Dar es Salaam. East Cent Afr j surg (Online).

[44] Ndiaye M, Dieng PN, Diop M, Sy MH, Diene JF, Pouye I (1995). [Closed traumas of the thorax. Assessment of two years activity at the Dakar Trauma Center]. Ann Chir.

[45] Sawa J, Green RS, Thoma B, Erdogan M, Davis PJ (2018). Risk factors for adverse outcomes in older adults with blunt chest trauma: a systematic review. Can J Emerg Med.

[46] LoCicero J 3rd, Mattox KL. Epidemiology of chest trauma. Surg Clin North Am. 1989;69(1):15–9. 10.1016/s0039-6109(16)44730-4 2911786

[47] Yadollahi M, Arabi AH, Mahmoudi A, Zamani M, Farahmand M. Blunt thoracic injury mortality and clinical presentation. Trauma Monthly. 2018;23(4).

[48] Battle CE, Evans PA (2015). Predictors of mortality in patients with flail chest: a systematic review. Emerg Med J.

[49] Battle CE, Hutchings H, Evans PA (2012). Risk factors that predict mortality in patients with blunt chest wall trauma: a systematic review and meta-analysis. Injury.

[50] Dangisso SS (2023). Effect of human factors on Road Traffic accidents (RTAs): the case of Hawassa City, SNRS, Ethiopia. PanAfrican J Gov Dev (PJGD).

[51] Nyadera IN, Osedo C (2023). Civil war between the Ethiopian Government and the Tigray people’s Liberation Front: a challenge to implement the responsibility to protect Doctrine. Afr J Confl Resolution.

[52] Turkson E, Oduro AD, Baffour PT, Quartey P (2023). Regional integration and non-tariff barriers to intra‐sub‐Saharan Africa trade. World Econ.

[53] Kagochi J, Durmaz N (2018). Assessing RTAs inter-regional trade enhancement in Sub-saharan Africa. Cogent Econ Finance.

