# Supplementary material for: Mortality of traumatic chest injury and its predictors across sub-saharan Africa: systematic review and meta-analysis, 2024
Source: BMC Emerg Med. 2024 Feb 27;24:32. doi: 10.1186/s12873-024-00951-w (PMC10900610; doi:10.1186/s12873-024-00951-w)
Supplement: Supplementary file 1 — Supplementary Material 1: Newcastle-Ottawa Scale adapted for cross-sectional studies for mortality of traumatic chest injurt [file 12873_2024_951_MOESM1_ESM.docx]

**Newcastle-Ottawa Scale adapted for cross-sectional studies**

**Selection:**

**1. Representativeness of the sample:**

a. Truly representative of the average in the target population. * (all subjects or random sampling)

b. Somewhat representative of the average in the target group. * (non-random sampling)

c. Selected group of users/convenience sample.

d. No description of the derivation of the included subjects.

**2. Sample size:**

a. Justified and satisfactory (including sample size calculation) *

b. Not justified.

c. No information provided

**3. Non-respondents:**

a. Proportion of target sample recruited attains pre-specified target or basic summary of non-respondent characteristics in sampling frame recorded. *

b. Unsatisfactory recruitment rate, no summary data on non-respondents.

c. No information provided

**4. Ascertainment of the exposure (risk factor):**

a. Vaccine records/vaccine registry/clinic registers/hospital records only. **

b. Parental or personal recall and vaccine/hospital records. *

c. Parental/personal recall only.

**Comparability: (Maximum 2 stars)**

**1. Comparability of subjects in different outcome groups on the basis of design or analysis. Confounding factors controlled.**

a. Data/ results adjusted for relevant predictors/risk factors/confounders e.g. age, sex, time since vaccination, etc. **

b. Data/results not adjusted for all relevant confounders/risk factors/information not provided.

**Outcome:**

**1. Assessment of outcome:**

a. Independent blind assessment using objective validated laboratory methods. **

b. Unblended assessment using objective validated laboratory methods. **

c. Used non-standard or non-validated laboratory methods with gold standard. *

d. No description/non-standard laboratory methods used.

**2. Statistical test:**

a. Statistical test used to analyse the data clearly described, appropriate and measures of association presented including confidence intervals and probability level (p value). *

b. Statistical test not appropriate, not described or incomplete.

**Cross-sectional Studies:**

**Very Good Studies: 9-10 points**

Good Studies: 7-8 points

Satisfactory Studies: 5-6 points

Unsatisfactory Studies: 0 to 4 points

This scale has been adapted from the Newcastle-Ottawa Quality Assessment Scale for cohort studies to provide quality assessment of cross sectional studies^12^.

*1. Herzog R, Álvarez-Pasquin M, Díaz C, Del Barrio JL, Estrada JM, Gil Á. Are healthcare workers’ intentions to vaccinate related to their knowledge, beliefs and attitudes? A systematic review. BMC public health. 2013 Dec;13(1):1-7.*

*2. Stang A: Critical evaluation of the Newcastle-Ottawa scale for the assessment of the quality of nonrandomized studies in meta-analyses. European journal of epidemiology 2010, 25(9):603-605.*

**Assessment Result**s

**Note that**

- **No star**

*** 1 point**

**** 2 points**

**Articles to assess vitamin A deficiency**

| Id | Studies | Representativeness of the sample (*) | Sample size (*) | Non-respondents (*) | Ascertainment of the exposure (**, *) | Comparability  (**) | Assessment of the outcome (**, *) | Statistical test  (*) | Total |
| --- | --- | --- | --- | --- | --- | --- | --- | --- | --- |
| 1 | Ararso Baru et.al 2020 | * | * | * | * | * | * | * | 8 |
| 2 | N. Ali and B. M. Gali et.al2004 | * | * | * | * | - | * | ** | 6 |
| 3 | Chijioke H Anyanwu et.al1981 | * | * | * | * | * | * | * | 7 |
| 4 | Ndiaye M  et.al1995 | * | * | * | * | * | * | * | 7 |
| 5 | Elias Degiannis et.al2006 | * | * | * | * | - | * | * | 6 |
| 6 | Eyo E Ekpe et.al2014 | * | * | * | * | * | - | * | 6 |
| 7 | EKPE.Ee.et.al 2018 | * | * | * | ** | ** | * | * | 9 |
| 8 | Seyoum Kassa et.al2023 | * | * | * | ** | - | ** | * | 8 |
| 9 | V Kong et.al2022 | - | - | * | * | * | * | ** | 6 |
| 10 | Monafisha K Lema2011 | * | * | * | * | * | * | * | 7 |
| 11 | F.A. Massaga et.al2010 | * | * | * | * | * | * | - | 6 |
| 12 | Elias Mdumaa et.al 2023 | * | * | * | * | * | * | - | 6 |
| 13 | ALAIN CHICHOM MEFIRE2010 | * | * | * | ** | * | * | * | 8 |
| 14 | M.A.Misauno et.al2007 | * | * | * | * | * | * | * | 7 |
| 15 | Abubeker Eshetu Yimam et.al2021 | * | * | * | ** | ** | ** | * | 9 |
| 16 | Addisu Taye et.al2022 | * | * | * | ** | ** | ** | * | 10 |
| 17 | Ogunrombi A.B. et.al2012 | * | * | * | * | * | * | - | 6 |
| 18 | Kelechi et.al 2015 | * | * | * | * | * | * | * | 7 |
| 19 | SD Peter et.al2021 | - | * | * | * | * | * | * | 6 |
| 20 | Mohammed Saeed et.al 2015 | - | * | * | * | * | * | * | 6 |
| 21 | OKUGBO et.al 2012 | - | * | * | * | * | * | * | 6 |
