# Supplementary material for: Mortality of traumatic chest injury and its predictors across sub-saharan Africa: systematic review and meta-analysis, 2024
Source: BMC Emerg Med. 2024 Feb 27;24:32. doi: 10.1186/s12873-024-00951-w (PMC10900610; doi:10.1186/s12873-024-00951-w)
Supplement: Supplementary file 2 — Supplementary Material 2: PRISMA 2020 flow diagram for new systematic reviews which included searches of databases and registers only [file 12873_2024_951_MOESM2_ESM.docx]

*Consider, if feasible to do so, reporting the number of records identified from each database or register searched (rather than the total number across all databases/registers).

**If automation tools were used, indicate how many records were excluded by a human and how many were excluded by automation tools.

*From:*  Page MJ, McKenzie JE, Bossuyt PM, Boutron I, Hoffmann TC, Mulrow CD, et al. The PRISMA 2020 statement: an updated guideline for reporting systematic reviews. BMJ 2021;372:n71. doi: 10.1136/bmj.n71

For more information, visit: <http://www.prisma-statement.org/>
